# The influence of nutritional habits, body mass index and intestinal microbiota in mastocytosis on clinical symptoms using conventional culture and next generation sequencing

**DOI:** 10.1002/clt2.12310

**Published:** 2024-01-13

**Authors:** Ewelina Harcęko‐Zielińska, Marek Niedoszytko, Aleksandra Górska, Sylwia Małgorzewicz, Marta Gruchała‐Niedoszytko, Marek Bronk, Slawomir Dąbrowski, Marta Chełminska, Ewa Jassem

**Affiliations:** ^1^ Department of Allergology Medical University of Gdańsk Gdańsk Poland; ^2^ Department of Clinical Nutrition Medical University of Gdańsk Gdańsk Poland; ^3^ Laboratory of Clinical Microbiology Gdańsk Poland; ^4^ A&A Biotechnology Gdynia Poland

**Keywords:** BMI, eating disorders, mastocytosis, microbiome

## Abstract

**Background:**

Mastocytosis is a rare neoplastic disease of the bone marrow associated with the proliferation and accumulation of mast cells in various internal organs, including the gastrointestinal tract. There are few studies describing the gut microbiome of patients with mastocytosis using next generation sequencing supported using traditional culture methods. The aims of the study were, firstly, the determination of nutrition habits, composition of the intestinal microflora and BMI in mastocytosis, and secondly, analysis of mastocytosis severity and symptoms depending on the composition of the intestinal microflora.

**Methods:**

The study included 47 patients with indolent systemic mastocytosis and 18 healthy controls. All participants gave their informed consent to participate in the study. The study consisted of 3 parts: I‐clinical assessment, II ‐ examination of the intestinal microflora using the biochemical method, III ‐ 16S rRNA sequencing.

**Results:**

The nutrition habits and BMI of mastocytosis patients were similar to controls; however, most patients with mastocytosis had a low dietary vitamin and mineral content. As many as 94.5% of patients had too little fiber intake and mineral content. The most common cause of the abnormal stool test result with traditional culture was a titer of *E*. *coli* <10^6^. The low richness of microbiota species indicated by the Simpson index was observed in mastocytosis, *p* = 0.04. There were no significant differences in the composition of the intestinal microflora depending on the type of mastocytosis; however, the tryptase level correlated with the amount of Suterella, Barnesiellaceae, Eubacterium, Odoribacter, and Anaerostipes.

**Conclusions:**

The nutritional habits and BMI of mastocytosis patients are similar to the general population, except for too little fiber intake and mineral content. The gastrointestinal symptoms of mastocytosis patients may be related to the low richness of microbiota species and the amount of Suterella, Barnesiellaceae, Eubacterium, Odoribacter, Anaerostipes, which correlated with tryptase levels.

## 1 KEY MESSAGE

Mastocytosis is a rare (accounting for less than 0.01% of the general population) group of neoplastic diseases of the bone marrow associated with the growth of mast cells and their accumulation in one or more organs, such as the skin, mucous membranes, liver, spleen, or bone marrow.^1^ About 80% of patients report gastrointestinal complaints, mainly disturbances in the rhythm of bowel movements.^2^ Histamine stimulates the secretion of stomach acid, which increases the risk of developing gastric ulcer and intestinal ulceration. The etiology of frequent diarrhea in patients with mastocytosis remains unknown. Studies suggest an impact of mast cell infiltration in the intestinal wall and the abnormality of the small intestine mucosa itself, which may lead to malabsorption and weight loss.^3^

The human microbiome consists of 500–1000 different microorganisms with a total biomass of approximately 1.5 kg^4^. Live cells of a fully developed intestinal microflora contain genetic information, which, according to preliminary findings, exceeds the number of host cells by up to a hundred times.^4^, ^5^ According to updated data from 2016, the ratio of bacterial cells to human host cells is closer to 1: 1.^6^

The diet is an important factor in modifying the intestinal microbiome. A high‐fiber diet plays a key role in the manifestation of diseases related to excessive mast cell proliferation. Short‐chain fatty acids—SCFAs produced by individual bacteria can inhibit mast cell degranulation at the level of the MAPK signaling pathway.^7^ Little is known about the role of the gut microflora in the pathogenesis of this disease.^8^ Due to the rapid technological progress and the constantly expanding knowledge in this subject, this is an exciting time in the study of the human microbiome, which may become an important step in the development of personalized therapy focused on prevention, health maintenance or treatment with a relatively low risk of adverse effects.^9^, ^10^ We found no clinical studies on mastocystosis and microbiome in the PubMed database. The observations made so far have focused mainly on the role of dietary fiber and its metabolites in the pathophysiology of mast cell diseases.^10^

The aims of the study were the determination of nutrition habits, composition of the intestinal microflora, and BMI in mastocytosis; secondly, the analysis of the mastocytosis severity and symptoms depending on the composition of the intestinal microflora.

## 2 METHODS

The study included 47 patients with mastocytosis (24 women, 24 men) with mean age in the 50 range (26–72) years. Systemic mastocytosis with skin lesions was diagnosed in 33 patients. Median tryptase was 43.6 ng/mL (range 7.71–288). The mean BMI was 28 range (17.78–41.02). The control group consisted of 18 healthy volunteers (10 women, 8 men; aged 32–70; the mean age was *M* = 50.89). BMI ranged from 19.16 to 32.03, and its mean value was 26.14. All participants gave their informed consent to participate in the study.

No statistically significant differences were found between patients with mastocytosis and the control group in terms of age, sex, BMI, and the occurrence of food allergies and hypersensitivity. The exclusion criteria were pregnancy, neoplastic diseases, heart, kidney and liver failure, confirmed infections of the digestive system, chronic inflammation of the intestines, probiotic intake and antibiotic therapy in the last month before collecting a stool sample. The study consisted of three parts. The first part consisted of the clinical assessment based on completed questionnaires on the most frequently reported symptoms, the FFQ questionnaire, and an interview in 24 h of nutritional memory. Secondly, the examination of the intestinal microflora with the method of traditional culture. Stool samples were collected from 45 people diagnosed with mastocytosis. In the third stage, 16 people with mastocytosis who participated in the previous stages of the study, were randomly selected for 16S rRNA sequencing using next generation sequencing (NGS). The control group consisted of 18 volunteers. The consent was given for the examination by the independent Bioethical Committee for Scientific Research of the MUG (approval no. NKBBN/374/2016.

The stool samples were collected by the patients into sterile containers in accordance with the instructions described in detail in Supplementary files. Briefly, the procedure consisted in collecting material from 8 different sites with a spatula to fill a 150 mL container to about ¾ of its height. The stool was cultured with the quantitative method at the Clinical Microbiology Laboratory of the University Clinical Hospital, where the material was immediately sent from the MUG Allergology Clinic or collected by the patient at home (stored in a refrigerator at 2–8°C) and delivered no later than 12 h before the planned medical visit. The obtained stool samples were marked with a special number and stored at ‐ 80°C until further determination.

### 2.1 Examination of the intestinal microflora

The method of serial dilutions was used to determine the number of cells of individual bacterial species in the tested stool samples. Assuming that the bacteria present in the titer of at least 10^5^ viable cells in 1 g of stool can modulate the properties of the intestinal microbiota and shape its influence on the functioning of the entire human body, bacteria were isolated from the stool suspension in dilutions from 10^5^ to 10^10^.

### 2.2 Examination of the intestinal microbiota using the NGS technique

DNA isolation from the collected samples was carried out at A&A Biotechnology in Gdynia. Genomic DNA was isolated by a modified method based on the Genomic Mini AX Bacteria + kit (A&A Biotechnology). Prior to the preparation of the V3‐V4 amplicon library, DNA eluates were checked for quantity and quality. Libraries for sequencing were prepared by random DNA fragmentation followed by ligation of the 5' and 3′ adapters. The adapter ligated fragments were then PCR amplified and gel purified. Libraries were prepared according to the 16S Metagenomic Sequencing Library Preparation Guidelines^11^ using Herculase II Fusion DNA Plymerase Nextera XT Index Kit V2. The quality of the library was checked according to the Illumina qPCR Quantification Protocol Guide.^12^ Libraries have been prepared by Macrogen. For sequencing, the material was transferred to the Macrogen Laboratory, IT and Business Headquarter & Support Center located in the Republic of Korea. Sequencing was performed using the Illumina MiSeq 2x300 bp platform.

Bioinformatics analysis was performed using CLC Genomic Workbench v. 12 (Qiagen) + Microbial Genomics Modul Plugin v. 4.1 (Qiagen). Data import for analysis and then their format was based on the read pairing (forward and reverse) and the removal of incorrect reads. The first stage of bioinformatics analysis was to remove duplicates and low‐quality readings (quality limit = 0.05). Then, the obtained sequences were assigned to appropriate taxonomic levels (i.e. genus, species) creating operational taxonomic units (Operational Taxonomic Units‐ OTU). The OTU alignment with the corresponding sequences was made using the Greengenes reference database.

Statistical analyzes were performed using the Statistica 13.3 TIBC software, IBM SPSS Statistics 25 and MS Excel spreadsheet. The level of statistical significance was *p* < 0.05. Statistical tests used in the analyzes are one‐way analysis of variance (ANOVA), Levene's test, post‐hoc NIR tests, Spearman and/or Pearson correlation, chi‐square test, Mann‐Whitney *U* test. Additional analysis of the gut microbiota was based on the determination of alpha and beta diversity using CLC Genomics Workbench version 22.0.

Alpha diversity describes species diversity and richness in a single sample. The Sh annon and Simpson index as well as Chao1 and Phylogenetic diversity metrics were used to assess alpha diversity, while the non‐parametric Kruskal‐Wallis test was used in the statistical analysis.

Beta diversity describes the differences in the taxonomic composition between samples. Principal coordinate analysis (PCoA) based on the matrix was used to assess beta diversity in Jaccard, Bray‐Curtis and Unifrac. Multivariate PERMANOVA analysis of variance was used to show differences in beta diversity, and threshold values ​​<0.05 were defined as statistically significant differences.

## 3 RESULTS

Results of the clinical assessment. The examined respiratory and digestive system symptoms, skin symptoms of mastocytosis were related to the frequency of consumption of the studied groups of products (containing glutamate, amines, salicylates, gluten, lactose). Skin symptoms include pruritus, erythroderma, flush, urticaria, angioedema, itchy lips, swelling of the eyelids, recurrent aphthous stomatitis, dry skin, and folliculitis. Gastrointestinal symptoms include abdominal cramps, nausea, vomiting immediately after eating, diarrhea, loose stools, constipation, flatulence, dyspepsia, and heartburn/burning in the esophagus.

The strongest correlations concerned gastrointestinal symptoms. A fairly strong correlation between the average consumption of the above‐mentioned product classes and the average total of reported symptoms was obtained (rho = 0.60; *p* = 0.000; *N* = 43). Such a correlation between the average consumption of these product classes and the reported symptoms was not statistically significant in the control group (rho = 0.44; *p* = 0.070; *N* = 18).

In order to determine the nutritional habits, data collected with the food frequency of consumption questionnaire (FFQ‐6) was used. FFQ‐6 questionnaire is a semi‐quantitative questionnaire validated for the Polish population concerning the frequency of consumption of 165 food products (food and beverages) at a specific time in the last month or year. When assessing the reproducibility of the results obtained using FFQ‐6, it was considered a valid measurement tool for assessing the frequency and amount of food consumed. The analysis showed no statistically significant differences in the studied groups (*p* < 0.05). The nutritional habits in patients with mastocytosis and in the control group were similar.

The results of the 24‐h diet diary were compared with the nutritional standards for the Polish population.^13^ It has been shown that the majority of patients with mastocytosis have low dietary vitamin and mineral content. All patients had an iodine and vitamin D content below the lower limit of normal, and most had insufficient dietary potassium, iron, calcium, magnesium, vitamin E, vitamin C and folate (Table 1). Particularly noteworthy is the fact that as many as 94.5% of patients had too little fiber.

**TABLE 1:** The content of vitamins and minerals in the diet of patients with mastocytosis the results of the dietary interview were related to the nutritional standards for the Polish population based on, Normy żywienia dla populacji Polski”, Mirosław Jarosz, Instytut Żywności I Żywienia, 2017.

	Norm	Mean	% Abnormal results
Carbohydrate [g]	>130	232.4	11
Protein [g]	*M* > 50, *F* > 41	M‐64.2 F‐ 65.2	M‐28.6, F‐0
Cholesterol [mg]	<300	327.7	66.67
Fiber [g]	25	17.2	94.5
Sodium [mg]	1500	1854.2	50
Potassium [mg]	3500	2786.6	99.9
Iron [mg]	M‐10, F‐18	M‐10.2, F‐14.4	M‐71.4, F‐72.7
Calcium [mg]	1000	562	88.9
Phosphorus [mg]	700	924.4	33.3
Iodine [ug]	150	20.8	100
Magnesium [mg]	M‐420, F‐320	M‐199, F‐299	M‐100, F‐63.6
Vitamin A [ug]	M‐620, F‐500	M‐769, F‐2553	M‐29, F‐90.9
Vitamin D [ug]	15	1.9	100
Vitamin E [mg]	M‐10, F‐8	M‐5.7, F‐6.9	100
Vitamin C [mg]	M‐90, F‐75	M‐63.2, F‐134.1	M‐62.5, F‐57.1
Vitamin B6 [mg]	1.3	1.6	11.1
Vitamin B12 [ug]	2.4	6	51.1
Folates [ug]	400	299	77.8

Abbreviations: F, female; M, man.

### 3.1 Results of the examination of the intestinal microflora by the inoculation method

Fecal culture was performed using the quantitative method in 45 patients with mastocytosis. Abnormal stool test results were obtained in 35 patients with mastocytosis. Based on literature data,^14^ the abnormal stool test result was defined as *Escherichia coli* <10^6,^ undetectable probiotic flora (i.e. below the adopted cut‐off point of bacterial detection) and the presence of pathogenic microorganisms. The most common cause of abnormal stool results was *E*. *coli* titer <10^6^. Among the microorganisms with high pathogenic potential in patients with mastocytosis, *Klebsiella pneumoniae*, *Clostridium perfringens*, *Clostridium baratii*, *Candida glabrata*, *and Candida albicans* were found.

### 3.2 The examination of the intestinal microbiota using the NGS technique

**FIGURE 1:**
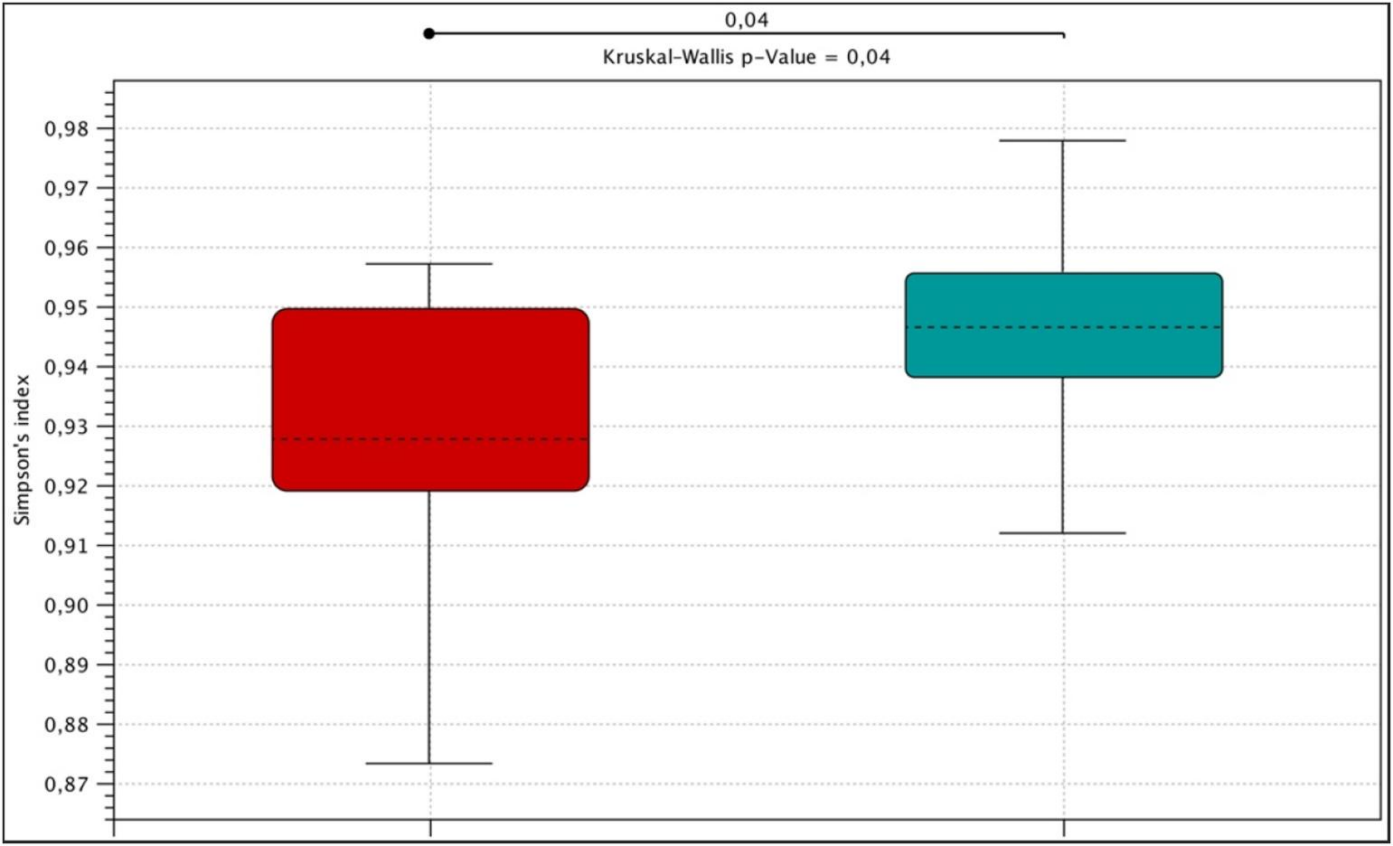
Alpha diversity expressed by the Simpson index in the control group (green), patients with mastocytosis (red).

The highest alfa diversity expressed by the Simpson index was observed in the control group and it was statistically significantly greater than in the group of patients with mastocytosis (*p* = 0.04, Kruskal‐Wallis). When analyzing species biodiversity expressed by the Shannon and Chao1 indexes, no statistically significant differences were found in the studied groups (Figure 1).

**FIGURE 2:**
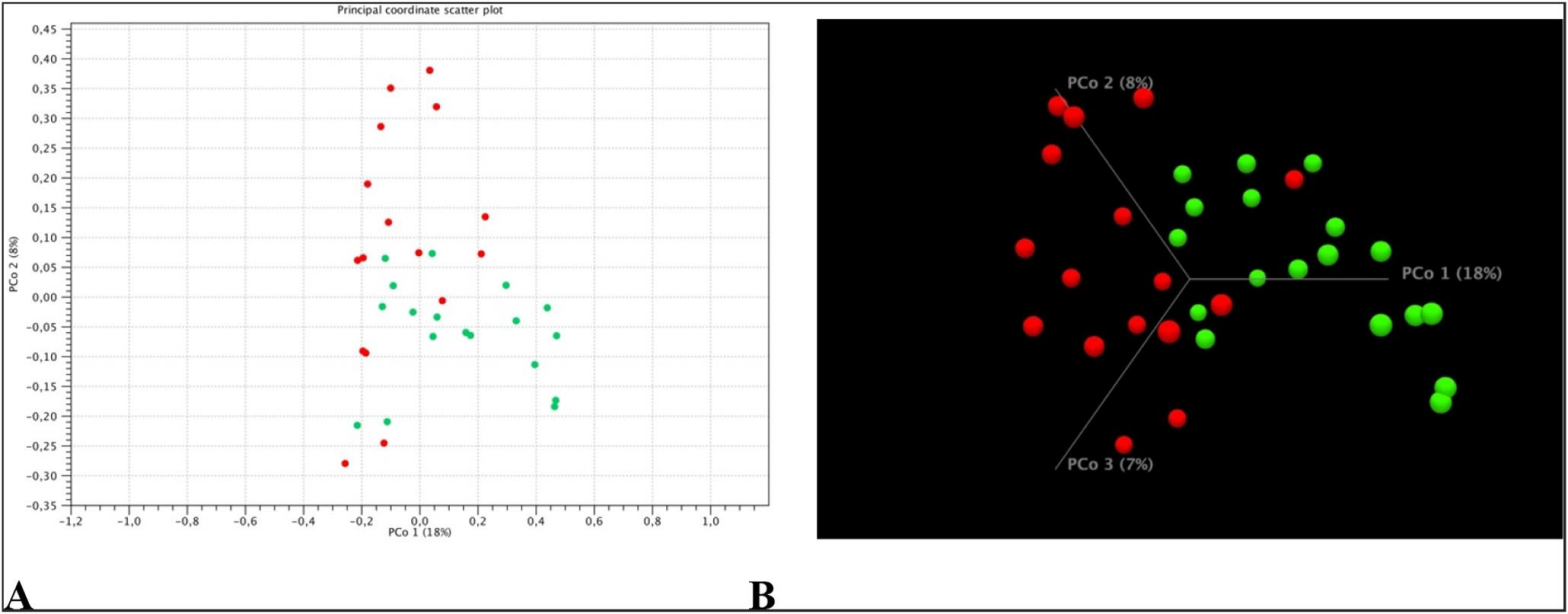
Beta diversity analysis in the study groups. Green points refer to the control group and red points to mastocytosis. Percentages in the axis descriptions represent the percentage of variation explained by the principal Principal coordinate analysis (PCoA) coordinates as measured using the Bray‐Curtis matrix. Figure (A) shows the 2D view, while figure (B) shows the beta diversity of the studied groups in 3D visualization. The results shown in the figure were calculated and visualized using the CLC Genomics Workbench 22.0 software.

Beta diversity was assessed using the matrix Jaccard, Bray‐Curtis and Unifrac. Based on the analysis PCoA showed a visible shift to the right of the control group (green points) relative to the group of patients with mastocytosis (red points) (Figure 2). Statistically significant differences in the composition of microbial communities were demonstrated by comparing the control group with the group of patients with mastocytosis (PERMAVOVA, Bray‐Curtis, *p* = 0.00011). Subsequent PERMANOVA analyzes using Jaccard, UniFrac weighted, Euclidean matrices also showed significant differences between the control group and mastocytosis (*p* = 0.00011 Jaccard, *p* = 0.00038 UniFrac weighted, *p* = 0.0001 Euclidean).

**FIGURE 3:**
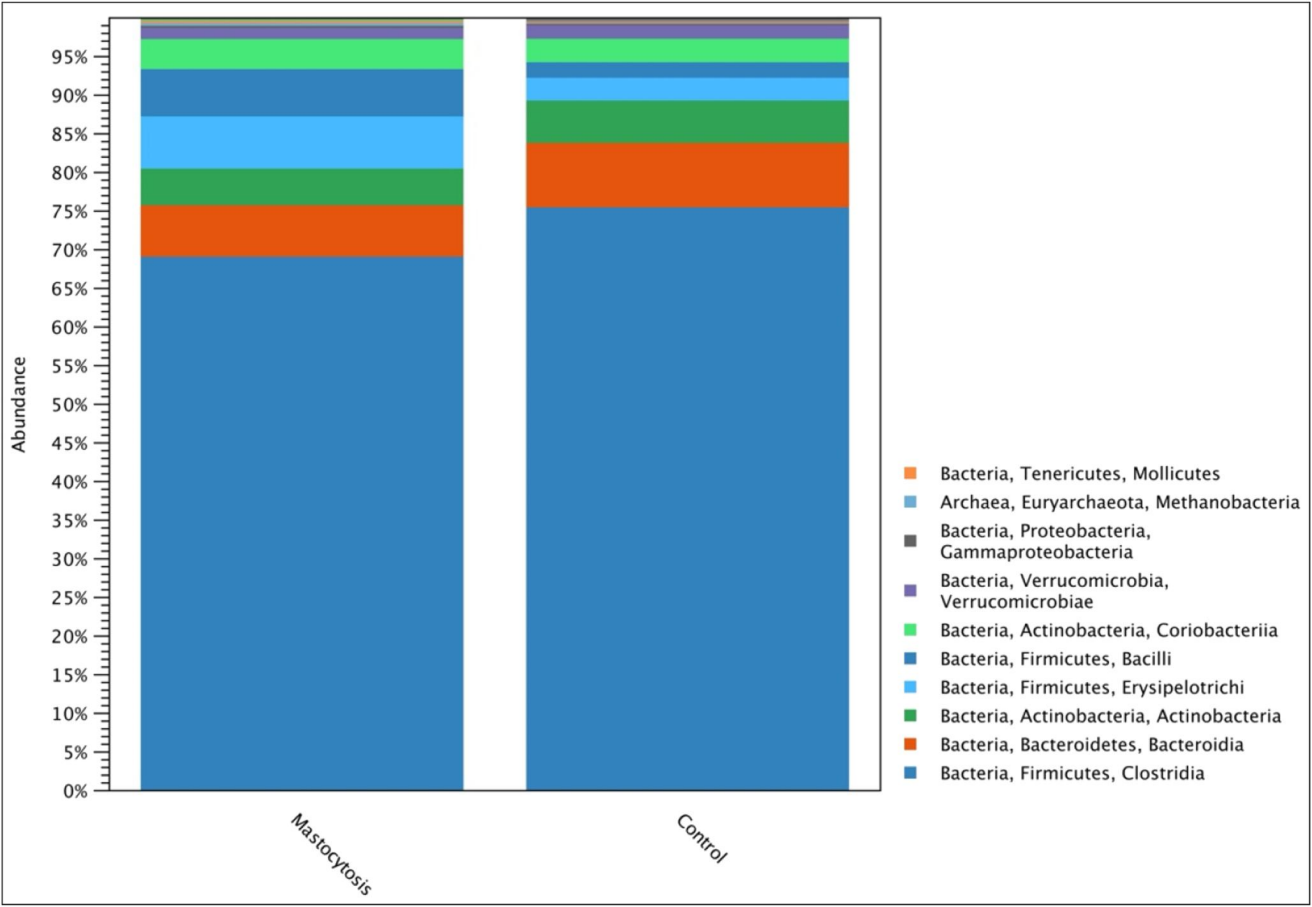
Relative microbial abundance at the class level in the study groups.

Firmicutes were the most numerous types of bacteria (phylum) in all studied groups, the relative abundance of which was expressed as a percentage, respectively: 81% in the control group and 82% in the group of patients with mastocytosis (Figure 3). *Bacteroidetes* constituted 8% in the control group and 7% in patients with mastocytosis, respectively. Another dominant type of bacteria was Actinobacteria, which accounted for 4.8% in patients with mastocytosis and 5.5% in the control group. There were statistically significant differences between mastocytosis and the control group (*p* = 0.04852) in the relative abundance of an unidentified genus belonging to the *Enterobacteriacea* family (*Gammaproteobacteria* group).

When analyzing the composition of the intestinal microbiota depending on the form of mastositis (ISM, BMM, SSM, MIS), no statistically significant differences were found in general bacterial profiles and in the biodiversity of microbial communities expressed by alpha and beta diversity.

**FIGURE 4:**
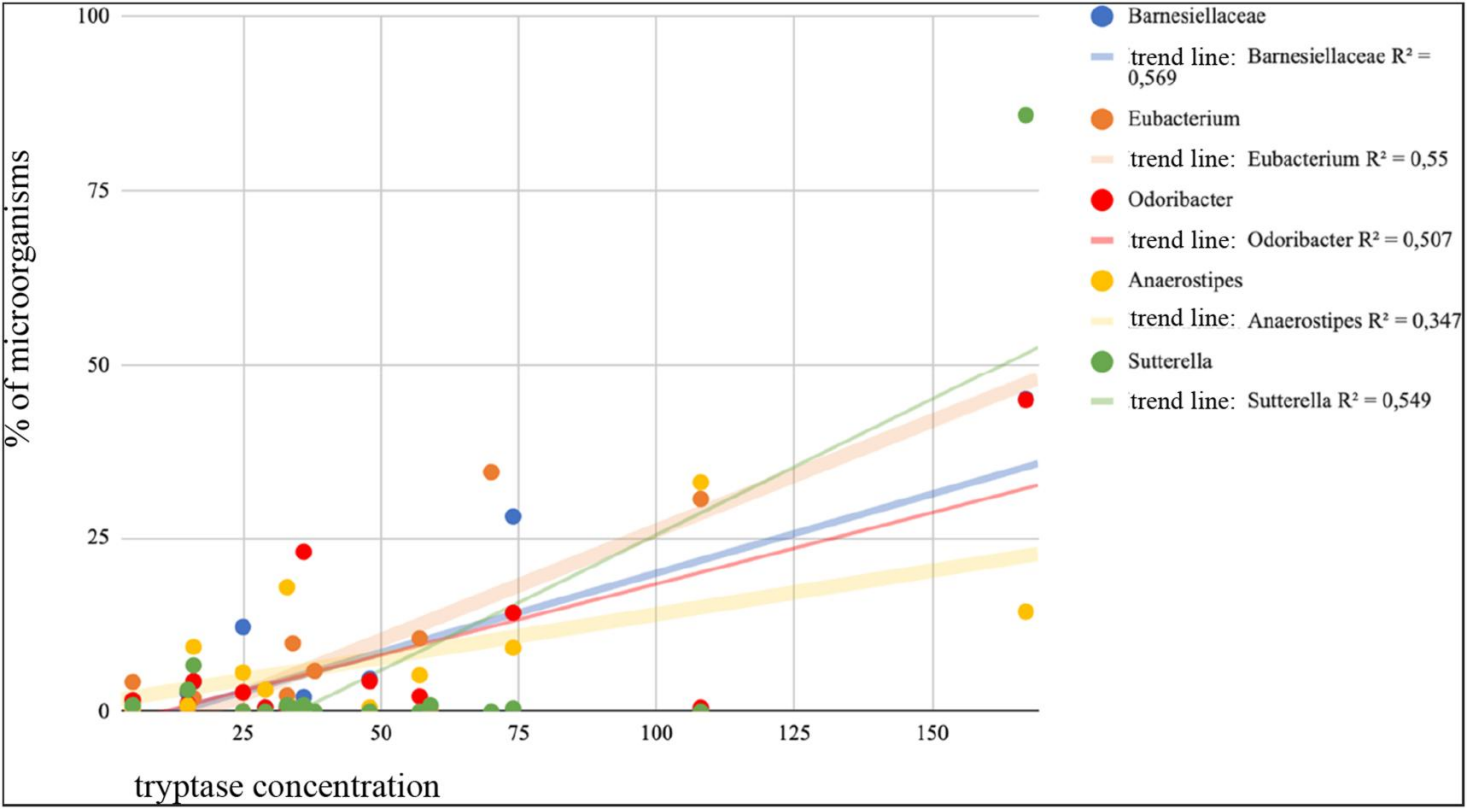
An analysis of the correlation between tryptase concentration and intestinal microorganisms in patients with mastocytosis.

In the group of patients with mastocytosis, a strong positive correlation between the concentration of mast cell tryptase and the bacteria of the genus Suterella, belonging to the type of Proteobacteria, was demonstrated, Figure 4. (Pearson *r* = 0.88, *p* = 0.0017) and the bacteria shown in the Table 2.

**TABLE 2:** Correlation between tryptase concentration and bacteria at the genus level in patients with mastocytosis.

Type	Genus	Pearson	*p*‐value
*Bacteroidetes*	*Barnesiellaceae* ^a^	0.75	0.0046
*Firmicutes*	*Eubacterium*	0.74	0.0037
*Bacteroidetes*	*Odoribacter*	0.71	0.0094
*Firmicutes*	*Anaerostipes*	0.59	0.0343

^a^ Unidentified genus belonging to the family Barnesiellaceae.

## 4 DISCUSSION

This study showed that the incidence of food intolerance and allergy was similar in patients with mastocytosis and in the control group. The above observations are consistent with the reports of Jarkvist et al., who showed that the incidence of food intolerance in patients with mastocytosis is similar to that in the general population.^15^ The dietary interview conducted on the basis of the 24‐h nutritional memory clearly showed that 94.5% of patients with mastocytosis have insufficient dietary fiber content. While there is evidence in the available literature that the host genotype affects microbiota differentiation, diet is believed to be a key factor in shaping the gut microflora.^16^, ^17^ Experimental studies in mice showed that dietary changes explained as much as 57% of the total variation in the structure of the gut microbiota, while the genetic basis was responsible for only 12% of the differences.^18^ Based on previous researches, it has been established that a plant‐based diet rich in fats or fiber can promote specific bacterial profiles.^19^, ^20^ For example, a high‐fat, low‐fiber diet leads to an increase in the ratio of Firmicutes to Bacteroidetes.^7^ Indeed, this study showed a higher percentage of Firmicutes and a lower percentage of Bacteroidetes in patients with mastocytosis. Folkerts et al. suggested that a low‐fiber Western European diet may contribute to a greater manifestation of allergic diseases.^7^, ^21^ The arguments of the researchers are confirmed by the parallel increase in the incidence of allergic diseases to the decreased consumption of fiber in developed countries. Regulating the mast cell activity with a high‐fiber diet is therefore a promising direction for new therapeutic strategies in patients with mastocytosis that we believe should be considered.

As expected, the highest alpha‐diversity expressed by the Simpson index was observed in the control group and it was statistically significantly greater than in the group of patients with mastocytosis. Greater richness and diversity in the gut microbiome is believed to be more desirable and associated with health.^22^ On the other hand, when analyzing beta diversity on the basis of PCoA, a visible shift to the right on the horizontal axis of the control group in relation to patients with mastocytosis was shown. The above results indicate differences in beta diversity defined as differences in the taxonomic composition between samples (mastocytosis vs. control group). The above observations may provide evidence of differences in the general structure of the intestinal microbiome in patients with mastocytosis and in the control group, resulting from the mechanism of allergy. This observation requires further research.

When analyzing the quantitative composition of individual types of bacteria, 5 types of bacteria dominated in the control group: Firmicutes, Bacteroidetes, Actinobacteria, Proteobacteria and *Verrrucomicrobia*, which is consistent with the previous findings on the intestinal microbiome of a healthy human.^20^, ^23^ In the sequencing process, patients with mastocytosis showed lower abundance of bacteria with beneficial effects for the host: *Faecalibacterium prausnitzii*, *Lachnospira and Roseburia*.*Faecalibacterium prausnitzii* are one of the most important producers of butyrate, the main source of energy for colonocytes. The anti‐inflammatory effect of butyric acid in the intestines consists of inhibiting the activity of the transcription factor NF‐kB, reducing interferon gamma IFN‐γ and increased PPARγ activity. In addition to its anti‐inflammatory effect, *Faecalibacterium prausnitzii* has a positive effect on the permeability of the intestines, strengthening the intestinal barrier.^24^ Earlier reports showed reduced abundance of *Faecalibacterium prausnitzii* in asthma, Crohn's disease, obesity and depressive disorders.^24^, ^25^, ^26^ *Roseburia* strengthens the intestinal barrier function and is an important producer of SCFA short‐chain fatty acids.^27^ *Lachnospira*, in addition to its ability to produce butyric acid, can also produce acetic acid, which, by affecting Treg cells, inhibited the allergic inflammatory reaction of the airways in a mouse model.^28^

The analysis of the commensal microflora of mastocytosis patients showed that *Escherichia coli* titer was below <10^6^. Furthermore, the studied *Escherichia coli* belongs to an extremely diverse species, most of which are non‐pathogenic. Many beneficial functions of E.coli living in the large intestine have been described; apart from participation in the synthesis of vitamins, its immunomodulatory properties in the host organism are also mentioned. According to some researchers, the lower abundance of non‐pathogenic *E*. *coli* strains is associated with allergic diseases.^29^, ^30^ Pang et al. It has been demonstrated that oral administration to mice of a non‐pathogenic strain of *E*. *coli* resulted in a statistically significant reduction of allergic symptoms in both the upper and lower respiratory tract.^30^ The mechanism of suppressing the allergic reaction consisted of reducing the production of pro‐inflammatory Th‐2 lymphocytes cytokines and increasing the activity of Treg lymphocytes secreting the anti‐inflammatory IL‐10.^30^ The researchers suggest that the above observations are consistent with the current “hygiene theory” and that the reduction of the commensal flora is disadvantageous in terms of achieving immune tolerance.

Commensal and probiotic bacteria that make up the intestinal microbiome are involved in the fermentation of fiber, the products of which are SCFA: acetate, propionate, butyrate.^31^, ^32^, ^33^ Diakos et al. were the first to provide evidence that butyrate can inhibit mast cell degranulation and reduce the production of tumor necrosis factor TNF‐alpha.^9^ Wang et al. confirmed the beneficial effect of butyrate on the degranulation of mast cells and the reduction of the production of pro‐inflammatory cytokines.^10^ Butyrate may regulate the function of mast cells through secondary pathways mediated by group 2 innate lymphoid cells (ILC2) 70.71. When subsequent observations bring hope for the possibility of using probiotic strains for specific prophylactic or therapeutic purposes in diseases related to the activation of mast cells, a question arises regarding the composition of probiotic microflora in this group of patients. To our knowledge, this is the first study to characterize the gut microbiome of people with mastocytosis using the NGS method, supported using traditional culture methods.

This work is one of the first studies characterizing the intestinal microbes in patients with mastocytosis. It has been shown that patients with systemic mastocytosis differ significantly not only in the overall structure of the gut microbiome expressed in alpha and beta diversity from healthy individuals, but also have a unique gut microbial profile. This is confirmed by previous reports of other researchers.^34^ The dominant types of bacteria were *Firmicutes*, *Bacteroidetes*, *Actinobacteria*, *Verrucomicrobia* and *Proteobacteria*. 373 operational taxonomic units unique for mastocytosis were found, which differed significantly from the healthy control group. In patients with mastocytosis, statistically significant differences in the abundance of bacteria belonging to the family *Erysipelotrichaceae* have been described. Although these microorganisms have been described relatively recently by Verbarg et al., there is a growing number of reports documenting the role of *Erysipelotrichaceae* in inflammatory diseases of the gastrointestinal tract.^35^ A greater number of this family of bacteria was found in patients with colorectal cancer.^36^ Changes in the abundance of *Erysipelotrichacea* were also observed in patients with inflammatory bowel disease.^37^ In contrast, Spencer et al provided strong evidence for the association of *Erysipelotrichacea* with host lipid disorders. The researchers showed that *Erysipelotrichi* and *Gammaproteobacteria* were directly related to choline deficiency‐induced steatosis. In this study, it was observed that the percentage of *Erysipelotrichi*it was even greater in patients with mastocytosis who reported abdominal pain. In subsequent studies, it would be interesting to establish the effect *Erysipelotrichacea*on lipid metabolism and inflammation of the gastrointestinal tract in this group of patients. The present study showed no statistically significant differences between the results of microbial stool culture and the concentration of mast cell tryptase. However, in the correlation analysis, the results obtained from bacterial sequencing of 16S rRNA showed a strong positive correlation of tryptase concentration with bacteria at the level of the genus: *Suterella*, *Eubacterium*, *Odoribacter*, and *Anaerostipes*. There is evidence that tryptase affects the differentiation of fibroblasts, causing fibrotic changes in the intestines.^38^ The above observations clearly show that in future studies of the intestinal microbiota one should look for bacteria characteristic of mastocytosis at the level of the genus, and not only refer to general changes.

## 5 CONCLUSION

The nutrition habits and BMI of mastocytosis patients are similar to the general population, except for too little fiber intake and mineral content. The gastrointestinal symptoms of mastocytosis patients may be related to the low richness of microbiota species and the amount of Suterella, Barnesiellaceae, Eubacterium, Odoribacter, and Anaerostipes, which correlated with tryptase levels.

## AUTHOR CONTRIBUTIONS

**Ewelina Harcęko‐Zielińska**: Conceptualization (equal); data curation (equal); formal analysis (equal); investigation (equal); methodology (equal); project administration (equal); resources (equal); software (equal); supervision (equal); validation (equal); writing – original draft (equal); writing – review & editing (equal). **Marek Niedoszytko**: Conceptualization (equal); data curation (equal); formal analysis (equal); funding acquisition (equal); investigation (equal); methodology (equal); project administration (equal); resources (equal); supervision (equal); validation (equal); writing – original draft (equal); writing – review & editing (equal). **Aleksandra Górska**: Conceptualization (equal); data curation (equal); formal analysis (equal); funding acquisition (equal); project administration (equal); resources (equal); supervision (equal); writing – review & editing (equal). **Sylwia Małgorzewicz**: Conceptualization (equal); data curation (equal); methodology (equal); resources (equal); supervision (equal); writing – review & editing (equal). **Marta Gruchała‐Niedoszytko**: Conceptualization (equal); data curation (equal); formal analysis (equal); resources (equal); writing – original draft (equal); writing – review & editing (equal). **Marek Bronk**: Data Curation (equal); investigation (equal); methodology (equal); resources (equal); software (equal); supervision (equal); validation (equal); writing – review & editing (equal). **Sławomir Dąbrowski**: Data Curation (equal); formal analysis (equal); investigation (equal); methodology (equal); resources (equal); software (equal); supervision (equal); validation (equal). **Marta Chełmińska**: Project Administration (equal); supervision (equal); writing – review & editing (equal). **Ewa Jassem**: Funding acquisition (equal); project administration (equal); supervision (equal); writing – review & editing (equal).

## CONFLICT OF INTEREST STATEMENT

The authors declare no conflicts of interest.

## Supporting information

Supporting Information S1

## Data Availability

Data available on request from the authors.
