# Supplementary material for: The influence of nutritional habits, body mass index and intestinal microbiota in mastocytosis on clinical symptoms using conventional culture and next generation sequencing
Source: Clin Transl Allergy. 2024 Jan 13;14(1):e12310. doi: 10.1002/clt2.12310 (PMC10787583; doi:10.1002/clt2.12310)
Supplement: Supplementary file 1 — Supporting Information S1 [file CLT2-14-e12310-s001.docx]

Supplementary Files


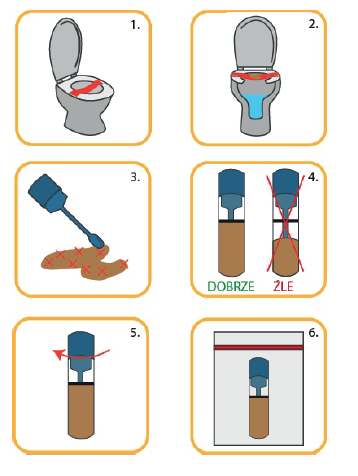


1. Stool collection instructions

(1,2) Stick a strip of paper on the flap.

(3) Using a spatula, collect faeces from eight different sites.

(4). Fill the container with feces up to the line marked.

(5) Close the container tightly and place it in a plastic bag.

2. Details for the verse 150-153

The frequency of occurrence of the symptom was scored as follows: 0 - never, 1 - sometimes, 2 - often, 3 - very often

In the next step, the consumption of products containing lactose, gluten, salicylates, amines and glutamate was assessed in the study groups, showing that the consumption of these products was similar.

However, when examining the relationship between product consumption and symptoms in patients with mastocytosis

It was shown that all the examined symptoms were related to the frequency of consumption of the examined groups of products. However, the strongest correlations were between these products and symptoms related to the gastrointestinal tract.

In the control group, fewer associations were found between the consumption of the studied groups of products and the reported symptoms than in patients with mastocytosis.

In the next step of the analysis, it was decided to check the correlation coefficients between the average occurrence of symptoms and the average consumption of products in the study groups. In the group of patients with mastocytosis, a fairly strong relationship effect was obtained between the average consumption of the above-mentioned classes of products and the average total of reported symptoms (rho = 0.60; p = 0.000; N = 43). However, in the control group, the relationship between the average consumption of the above-mentioned classes of products and the average total of reported symptoms was not statistically significant (rho = 0.44; p = 0.070; N = 18).

3. Details for the verse 189-191

The above results indicate differences in beta diversity defined as differences in taxonomic composition between samples (mastocytosis vs. control group). The above observations may provide evidence of differences in the general structure of the intestinal microbiome in patients with mastocytosis and in the control group, resulting from the mechanism of allergy. This observation requires further research.

4. Details for the verse 262-263

Assuming that a single colony is formed from one bacterial cell, the number of viable cells present in 1 gram of the tested faecal sample (bacterial titer) was determined using the following formula:

N = n x R x V

N - number of bacterial cells per gram of stool sample

n - number of bacterial colonies grown on the bacteriological medium in the plate.

R - dilution of the suspension from which the inoculation was made on the substrate (e.g. 10^5^, 10^6^, etc.)

V - the volume of suspension sown on the substrate as the denominator of the decimal fraction of the volume sown with 1 mL of suspension (in this case 100, because 1/100 mL was sown.).

Based on literature data, stool abnormalities were defined as Escherichia coli < 10^6^.
